# Utility and Feasibility of Transcutaneous Spinal Cord Stimulation for Patients With Incomplete SCI in Therapeutic Settings: A Review of Topic

**DOI:** 10.3389/fresc.2021.724003

**Published:** 2021-09-24

**Authors:** Rebecca Martin

**Affiliations:** ^1^International Center for Spinal Cord Injury, Kennedy Krieger Institute, Baltimore, MD, United States; ^2^Department of Physical Medicine and Rehabilitation, Johns Hopkins School of Medicine, Baltimore, MD, United States

**Keywords:** spinal cord stimulation, spinal cord injury, rehabilitation, neuromodulation, ambulation

## Abstract

Transcutaneous Spinal Cord Stimulation (TSCS) has been shown to enhance the excitability of spinal neural circuits. This excitation is associated with enhanced voluntary performance in patients with incomplete SCI (iSCI). Though there is much we do not know, combining this altered state of exciability with therapy has the potential to enhance the outcomes associated with activity-based interventions. It is a promising tool to augment the work being done in therapeutic settings with the potential to expedite recovery. There is, however, a lag in assimilating the science for clinical practice. This article will examine current literature related to the application of TSCS in combination with therapeutic interventions for motor recovery and aims to elucidate trends in waveform selection, duration and frequency, and combinatorial therapies that may inform clinical practice. With specific consideration for therapeutic settings, potential benefits, applications, and pitfalls for clinical use are considered. Finally, the next steps in research to move toward wider clinical utility are discussed.

## Introduction

The disruption of transmission of motor and sensory information associated with spinal cord injury (SCI) significantly impacts a person's ability to purposely move. Nearly 70% of new injuries each year are incomplete with incomplete tetraplegia as the fastest growing injury classification (1). Many of these patients will present with motor dysfunction (2). Recovery of hand and walking functions are a high priority among individuals incomplete SCI (iSCI) and a common target of physical therapy (3).

Compensation strategies used early in traditional rehabilitation provide patients with some level of independence, but focus primarily on strong reliable movements (2). These strategies ignore the potential for recovery of function and lack the input to the central nervous system (CNS) required to induce change (4). At best, traditional rehabilitation strategies artificially limit patients' functional status, at worst they lead to progressive loss of latent function and worsening disability over time (5). As patients with SCI are living longer and more active lives, there is a desire to return to more normal function thereby reducing secondary complications and accessibility barriers, which are costly in money, time, and quality of life. Driven by patient demands and emerging evidence on the recovery and repair of CNS damage, there is a rehabilitation paradigm shift toward return to pre-injury function (2, 6, 7). Activity-based therapy (ABT), which provides intensive, high repetition training, is considered the most effective intervention to improve walking function following iSCI (8–11). Providing high-volume, task-specific training to both improve the kinematics of walking and upregulate CNS activity, locomotor training has been shown to yield clinically meaningful improvements in gait speed, endurance, balance, and lower extremity strength (2, 12–14). However, even with these improvements, significant deficits in walking function persist and recovery of independent walking remains elusive for most patients with iSCI. Similarly, upper extremity interventions lack the consistency and durable changes associated with meaningful recovery.

The ABT evidence highlights the need for increased activation of the CNS to induce change in motor function (4, 11). Spinal cord stimulation was offered by researchers as an option to provide neuromodulatory input to potentiate gains achieved through physical rehabilitation interventions alone (15). Implanted lumbosacral epidural spinal cord stimulation (eSCS) (15–18), which acts by directly stimulating the dorsal nerve roots to increase the excitability of interneuronal networks involved in the control of locomotion, was explored first in animals and then human models (16, 18–20). Researchers demonstrated that lumbosacral epidural stimulation alone can facilitate reciprocal, step-like movements, and when used in combination with intensive locomotor training can lead to improved walking abilities (16, 20–23). Researchers have demonstrated that after long-term eSCS with training in chronic, complete SCI, recovery of volitional movement is possible, even in the absence of stimulation (24).

While impact of these improvements on quality of life for patients with SCI cannot be underestimated, the true functional impact and durability of these improvements remains to be seen. eSCS also carries some inherent risk, notably invasive surgical placement of the stimulator (18, 20, 21, 25, 26). Additionally, there is a large variability in the stimulation parameters and rehabilitation protocols reported post-implantation, ranging from 0 to 85 weeks of intensive training (16, 20, 27). Though all participants showed improvement in voluntary motor control, not all recovered durable walking function, nor sustained usage of the implant (16, 20, 24).

Transcutaneous spinal cord stimulation (TSCS) has the potential to impact CNS excitability and, when paired with training, create functional changes in patients with iSCI, that may be comparable to results of eSCS (28). TSCS represents a promising, clinically useful adjunct to existing physical rehabilitation interventions, without the risk and accessibility issues associated with surgical implantation for eSCS.

The purpose of this mini review is to elucidate trends and discuss the clinical relevance of TSCS as an adjunct to physical rehabilitation interventions. A PubMed search was conducted with the following search terms in all fields: transcutaneous spinal cord stimulation AND spinal cord injury AND rehabilitation. Articles were limited to the last 5 years (2016–2021) and English language. Articles that included exclusive examination of animal experiments, other diagnoses (ex: multiple sclerosis and cerebral palsy), and autonomic and non-voluntary functions (ex: bladder) were excluded. The discussion here is limited to studies with clinical relevance, as such, study of healthy persons and description of isolated neurophysiologic charges are also excluded. One hundred fifteen abstracts were screened, 13 articles were reviewed in full and are included here. Table 1 includes key study elements and a brief description of the results of each reference. This mini review aims to build upon the work of previous systematic reviews (41) of the topic by revealing trends meaningful for clinical settings.

**Table 1:** Key study elements and brief results.

**References**	**Design**	**N**	**SCI NL/AIS**	**Intervention**	Stimulation details^*^	**Results**
Gad et al. (29)	CR	1	T9 A	Training in EKSO robot +/- TSCS and fEmc	Electrodes at T11 (30 Hz) or Co1 (5 Hz), intensity to tolerance	pcEmc + fEmc improved voluntary effort and coordination during stepping. pcEmc alone resulted in more modest improvements. fEMC alone had no positive impact on stepping.
Freyvert et al. (30)	CCT	6	C2-C6 B	Grip strength exercises +/- TSCS and buspirone	Electrodes at C5, 5–30 Hz, 20–100 mA	pcEMc with or without buspirone improved grip force. UEMS, ARAT scores, and spasticity improved over the duration of the study.
Gad et al. (31)	CS	6	C4-C8 B, C	Grip strengthening and grasp and release training + TSCS	Electrodes at C3-C4 and C6-C7, 30 Hz with 10 kHz carrier frequency, 1 ms pulse duration, 10–250 mA	Subjects demonstrated greater grip force and activation of distal musculature with TSCS (~325%) and without TSCS following 8 training sessions (~225%). EMG shows inhibition of proximal UE muscles with multisite stimulation.
Inanici et al. (32)	CR	1	C3 D	UE interventions +/- TSCS	Electrodes at C3-C4 and C6-C7, 30 Hz with 10 kHz carrier frequency, 1 ms pulse duration, 80–120 mA	TSCS + therapy yielded improvements in strength, dexterity, and prehension, as reflected on the GRASSP, UEMS, and functional tasks. Gains were maintained during follow-up without stimulation.
Rath et al. (33)	CS	8	C4-C9 A, C	Motor tasks during sitting +/- TSCS	Electrodes at T11-T12 (30 Hz) and L1-L2 (15 Hz) with 10 kHz carrier frequency, 1 ms pulse duration, 10–140 mA	During TSCS, subjects were able to achieve a more erect posture and sustain wider perturbations, as compared to sitting without TSCS.
Sayenko et al. (34)	CCT	15	C4-T2 A, B, C	Standing exercises +/- TSCS	Electrodes at T11-T12 and L1-L2, 0.2–30 Hz with 10 kHz carrier frequency, 1 ms pulse duration, 10–150 mA	During TSCS, subjects were able to maintain upright standing with minimum to no stimulation. Seven subjects recovered independent standing with only intermittent UE support during stimulation. Without stimulation, none of the subjects could maintain standing without external support.
Alam et al. (35)	CR	1	C7, NR	Standing, treadmill walking, and LE strengthening + TSCS	Electrodes at T11 and L1, 0.5–30 Hz with 9.4 kHz carrier frequency, 100 μs-1 ms pulse duration, 20–120 mA	100 μs stimulation yielded more consistent muscle recruitment per EMG, as compared to 1 ms stimulation. After training, subject recovered volitional LE movement and functional skills (sit to stand, upright weight bearing). These gains were maintained 6 weeks after training and without stimulation.
McHugh et al. (36)	CS	10	C4-T9 C, D	Walking-based therapy + TSCS	Electrodes at T11-T12, biphasic symmetrical wave, 50 Hz, 1 ms pulse duration, 20–80 mA	Subjects demonstrated significant improvements in walking speed, endurance, and quality following 8 weeks of training. No subjects reported pain with stimulation. Some subjects reported improvement in bowel, bladder, and pain markers.
Meyer et al. (37)	CS	10	C3-T10 D	Ankle control exercises +/- TSCS	Electrodes at T11-T12, biphasic rectangular wave, 15/30/50 Hz, 1 ms, 15–70 mA	Immediate significant improvements in ankle motility were observed at 30 Hz, with suppression of pathological activity, assessed by polysynaptic spinal reflex. Non-significant improvements in walking speed were also observed.
Shapkova et al. (38)	CS	19	C5-T12 A, B, C	Exoskeleton walk training + TSCS	Electrodes at T12, monophasic square wave, 1/3/67 Hz, 0.5 ms, <70 mA	Ekoskeleton walk training with stimulation improved weight loading capacity and decreased gait asymmetry. Higher frequencies (67 Hz) had an antispasticity effect allowing independent walking. Subjects reported changes in proprioception, sensation, and paresthesias while walking with TSCS.
Zhang et al. (39)	CR	1	C5 A	UE interventions + TSCS	Electrodes at C3-C4 and C7-T1, 30 Hz with 10 kHz carrier frequency, 1 ms pulse duration, 15–50 mA	UE function (GRASSP, NRS, grip strength) improved after 18 sessions of task specific training with TSCS. These gains were maintained without stimulation at 3 months.
Estes et al. (40)	RCT	16	C1-T11 B, C, D	Locomotor Training +/- TSCS	Electrodes at T11-T12, biphasic symmetrical wave, 50 Hz. No pulse duration was reported, but the indicated device has a maximum output of 400 μs. Intensity is reported only as submotor.	Significant improvements in walking function (speed and symmetry) were observed in the LT+TSCS group. The control group did not show significant improvements. Neither group showed changes in spasticity, though large variations may have obscured change measurements. No subject reported stimulation-related pain limits to participation. TSCS was a useful and feasible adjunct to LT.
Inanici et al. (28)	CCT	6	C3-C5 B, C, D	UE interventions +/- TSCS	Electrodes above and below the LOI, 30 Hz with 10 kHz carrier frequency, 1 ms pulse duration, 40–90 mA	Intensive training with TSCS restored UE function (strength and prehension) better than training alone. Subjects also reported improvements in spasticity and autonomic functions. Gains were maintained at follow-up (3–6 months) without stimulation.

* *Stimulation details are reported here as they are in their respective studies. Detail and descriptions of stimulation vary greatly and all parameters were not available for all studies*.

*AIS, American Spinal Injury Association Impairment Scale; ARAT, Action Research Arm Test; CCT, cross-over clinical trial; CR, case report; CS, case series; fEmc, pharmacological enabling motor control; GRASSP, Graded Redefined Assessment of Strength, Sensibility, and Prehension; LE, lower extremity; LOI, level of injury; LT, locomotor training; N, sample size; NL, neurological level; NR, not reported; NRS, Neurorecovery Scale; pcEmc, painless transcutaneous electrical enabling motor control, stimulation is intended to enable task performance, avoiding direct muscle contraction; RCT, randomized control trial; UE, upper extremity; UEMS, upper extremity motor score*.

## What We Know

Via computational modeling and human EMG studies, non-invasive spinal cord stimulation has been shown to increase excitability of local spinal networks via dorsal root afferents with additional signal enhancement along the full length of the spinal cord (34, 42–45). This change in excitability capitalizes on functionally silent descending pathways to unmask and enhance voluntary movements of involved limbs (34, 44, 45). The priming of the nervous system offered via TSCS could augment existing physical rehabilitation interventions and has shown promise in many common therapeutic targets. TSCS, both in single sessions and repeated applications, is associated with improved standing postural control, gait kinematics, and upper extremity function. TSCS has also been demonstrated to have an impact on autonomic and non-voluntary functions (blood pressure regulation, bladder function, etc.) but those will not be discussed here (28, 34, 36, 46). Therapists should be aware that these may be consequences of interventions targeting motor recovery.

Early reports of TSCS demonstrate involuntary stepping with both single-session and repeated-exposure stimulation (45). Stimulation was delivered via electrodes over the T11/T12 or L1/L2 intervertebral space with anodes over bilateral iliac crest. The waveform consisted of 1 millisecond (msec) pulses with a frequency of 5–30 Hz, filled by a 10 kHz carrier frequency. Participants placed in a side-lying gravity eliminated position and stimulated as described exhibited oscillatory, step-like movements without voluntary effort. Participants demonstrated greater amplitude hip and knee oscillations when stimulation was combined with voluntary effort and with the addition of a second coccygeal stimulation site. Notably, this effect was demonstrated in uninjured, chronic complete and incomplete SCI. This work demonstrates that spinal neural circuits can be altered through spinal stimulation, supraspinal inputs (voluntary effort), and a combination of the two. This allows the field to shift from isolated stimulation to stimulation with training for recovery of voluntary effort.

### Lower Limb Applications

In 2018, 15 participants with chronic SCI demonstrated recovered ability to maintain upright standing with minimal to no external assistance, following TSCS and training (34). Stimulation paradigms matched those described above (45), notably with use of carrier frequency. Participants showed decreased motor activation threshold, increased lower extremity (LE) muscle activity via surface EMG, and improved weight acceptance during TSCS. Without stimulation, none of the participants were able to support themselves in upright. None of the participants reported pain associated with stimulation or adverse events during the course of the study. Participants reported changes in spasticity, proprioception, and mood, which were not quantified. The authors note the relative speed of skill acquisition, as compared to eSCS studies (18, 20), suggesting that TSCS may have a broader modulatory impact on neural networks and multisegmental projections.

Using the same stimulation parameters with task-specific training, benefits to trunk control (33) and ankle mobility (37) have been reported in participants with both complete and incomplete SCI. Participants improve performance with stimulation only and no maintenance of change without stimulation are reported. Here, again, the speed of skill acquisition is highlighted by the authors.

Exploration of walking functions with TSCS include a wide variety of interventions including over-ground, treadmill-based, and robotic-assisted studies. In a single case report (29), a participant with motor complete SCI underwent training in an exoskeleton with TSCS that included a carrier frequency. In a larger case series (38), participants were provided similar exoskeleton-based training with TSCS without a carrier frequency. The benefits reported were consistent across the two studies including improved voluntary control, coordination, and weight acceptance during stepping. In addition to the differing waveforms, authors of respective studies explored the impact of frequency on spasticity interrupting smooth gait. Higher frequencies were found to have the most benefit on spasticity (38).

Studies of over-ground and treadmill-based walk training with TSCS show similar improvements in gait (35, 36, 40). First, ten participants with iSCI received 23 sessions of TSCS with walking-based physical therapy (36). Here stimulation was delivered with a commercially available, clinically relevant stimulator using a biphasic waveform with 1 ms pulses at 50 Hz. Electrode placement was consistent with previous studies. Authors report that statistically significant improvements in walking speed, endurance, and quality, with changes exceeding individual test minimal clinically important difference (MCID) at or before sessions 18. Again, it is highlighted that TSCS yield functionally important improvements in shorter time frames than traditional models of care or with eSCS. As an adjunct to this work, another group published the results of an experiment comparing TSCS with locomotor training to sham stimulation with locomotor training (40). Biphasic stimulation was again provided with a commercially available stimulator. Participants receiving the experimental stimulation improved their walking speed and distance. No adverse events or protocol deviations due to pain were reported in either paper. Authors of both groups endorse the feasibility of TSCS in clinical settings. In a single case report (35), TSCS including a carrier frequency with the same electrode placement was applied during standing, treadmill walking, and strengthening activities. Authors report recovery of volitional movement and functional skills, which were maintained over 6 weeks without stimulation. The report of more durable changes indicates the addition of TSCS may contribute to recovery of function, rather than a transient state of hyperexcitability.

### Upper Limb Applications

Improvements in upper extremity (UE) function are also reported in relation to TSCS. In single-case reports (32, 39), case series (31) and prospective cohorts (28, 30) of subjects with motor incomplete SCI, sustained recovery of arm and hand movement is reported. In all of these reports stimulation was delivered in 1 ms pulses at 5–30 Hz, with a carrier frequency of 10 kHz. Electrode placement included one or more cervical intervertebral space, sometimes surrounding the level of injury. Anodes remain over the iliac crest. Interventions varied widely, as is common in UE exploration, with more focused training in grasp and release, as compared to other patterns of prehension. As with lower extremity and walking outcomes, the magnitude and speed of changes associated with TSCS, as compared to traditional rehabilitation is highlighted. Grip strength and functional dexterity show improvement with TSCS and, in several cases, improvements are shown to be durable over time without continued stimulation. TSCS is also reported to have a benefit on spasticity management in the UE (28).

## What We Don't Know

With all the evidence and apparent utility in relation to therapeutic targets, it stands to wonder why TSCS does not have greater clinical deployment. The first obvious issue is lack of consensus on stimulation parameters. Most studies use low frequencies (30–50 Hz) (36, 40) but some add a carrier frequency (34, 45), claiming this makes the stimulation pain free. Proponents claim the carrier frequency selectively blocks transmission of pain information and lowers tissue impedance for deeper penetration of stimulation (47). However, direct comparison of a traditional a symmetrical biphasic waveform and a waveform with carrier frequency found no significant difference in intensity necessary for motor activation and participants' subjective reports of discomfort was equal across the stimulation paradigms (48). Largely the field agrees that electrode placement determines current direction and consequently motor targets (i.e., cervical placements for UE interventions). Without large-scale controlled trials, it is difficult to pick the most efficacious stimulation parameters. As a somewhat related issue, identifying the best responders and the need for individual adjustments in stimulation parameters is a gap in our collective knowledge. Existing evidence suggests that TSCS may be more useful in iSCI, but perhaps it also has a role in screening or conditioning patients with complete injury prior to eSCS. The availability of appropriate stimulation devices may also be an issue for successful deployment into therapeutic settings. Few studies use commercially available, clinically relevant stimulators (36, 40). Additionally, longitudinal study is warranted to more fully glean the impact of TSCS. We don't yet understand if the changes associated with the intervention are long lasting, or if patients will need to use neuromodulatory inputs regularly, like a vitamin or charging a battery, or only as needed, like an orthotic for walking. Finally, there are always the limitations and barriers associated with clinical services, including time, training, and reimbursement.

## How to Close the Gap

Even with all the unknowns, the data in support of TSCS is compelling. Though very few studies have demonstrated the use of TSCS in clinical settings, they have made a case for its utility and feasibility, demonstrating it to be a low burden, low risk adjunct to existing interventions (36, 40). When looking at the studies discussed here in aggregate, a few commonalities can be identified. Successful TSCS applications include long pulse duration (0.5 μs to 1 ms) and moderate frequencies (30–50 Hz). Electrode placements targeting upper and lower extremities are well-defined. There is no conclusive evidence that the carrier frequency is necessary. Outcomes between studies with and without carrier frequency are similar and in direct comparison, subject tolerance was not impacted by the carrier frequency. Carrier frequencies are not readily available in clinical stimulators and so, in their absence, clinicians may opt to pursue TSCS with standard biphasic waveforms. For the evidence to be advanced and conclusive decisions on parameters to be made, well-controlled, larger sample studies are needed. Given the heterogeneity and recruitment challenges associated with SCI, this may require multiple centers to come together, agreeing on study design, stimulation parameters, and outcomes.

Finally, there is likely benefit in extending the study of TSCS into other populations. Work has begun in children with iSCI and cerebral palsy (CP) (49, 50). Reports of decreased spasticity lends TSCS to other neurological diagnoses, like stroke (51) and multiple sclerosis (MS) (52), where dysregulation in the CNS leads to dysfunction and atrophy of spinal neural networks. The more populations in which TSCS is demonstrated as useful, the more attention and funding it will draw, ultimately leading to better acceptance in therapeutic settings.

Increasingly, patients are acutely aware of these developing and advancing interventions and seek clinics willing to offer them. Companies are stepping in to fill the technology void and so the responsibility falls to the therapists to decide who and how and when.

## Author Contributions

RM was the sole author of this article and responsible for all the work herein.

## Conflict of Interest

RM receives research support from the Orokawa Foundation and Niche BioMedical.

## Publisher's Note

All claims expressed in this article are solely those of the authors and do not necessarily represent those of their affiliated organizations, or those of the publisher, the editors and the reviewers. Any product that may be evaluated in this article, or claim that may be made by its manufacturer, is not guaranteed or endorsed by the publisher.
